# Buffalo Lithia Springs

**Published:** 1879-05-20

**Authors:** Goodbridge A. Wilson

**Affiliations:** Sommerset, Granville Co., N.C.


### *THE MEDICAL PROPERTIES AND USES OF THE WATERS OF THE BUFFALO LITHIA SPRINGS OF MECKLENBURG COUNTY, VIRGINIA.*

BY GOODBRIDGE A. WILSON, M.D.

[Sommerset, Granville Co., N.C.]

The remedial power of these waters is now established beyond the reach of reasonable cavil. The traditional and abiding reputation given them by the jury of the vicinage, is fully vindicated by the more methodical trials of educated and scientific inquirers. Many medical experts have borne testimony, furnishing a body of experimental proofs which it would be folly to gainsay; proofs drawn from careful observations made in the sick chambers of homes, scattered to the breadth of the land, and without the well-recognized adjuvants of travel, change of air and scene, and diet, and social life, and freedom from all business cares.

These testimonies concede and attest curative powers over a wide range of pathological states, the very extent of this range giving to the candid objector his most rational excuse for the rejection of facts, as well established as any can be by human testimony.

On this ground, skepticism has been indulged of the remedial virtues of all mineral waters, and probably will continue as long as men will insist on estimating, gauging and subordinating the chemical combinations of nature's great laboratory to the artificial products of the coarser chemistry of their shops.

However inclined to such abstract reasoning, a truer philosophy will guide to a safer decision (on this, as on some other subjects) when it heeds the voices constantly proclaiming : "Whereas, I was blind, now I see."

An attempt to classify the properties of these waters, and enumerate the pathological states to which they have shown adaptability, is the design of this paper—and these necessarily involve some consideration of the *modus operandi.* It is designed for the professional reader, and within its limits can be but little else than suggestive.

And 1st the *physiological* action of these waters.

Any healthy person, capable of observing the functions of his body on their use, will note a marked exhilaration of spirits and exaltation of nerve force—as prompt as from use of a generous glass of wine—and most notably observed by those unaccustomed to the use of stimulants. There is also manifest increase in the force and frequency of the heart's action. In some, this exaltation of nerve force almost attains to a pathological state, being attended by cerebral pains and other nervous disquiets—but these latter seem to be promptly removed by the supervention of the second train of effects from these waters, viz : a notable increase in the secretions and excretions of all the glands of the body, and especially the great common emunctories—the skin and kidneys. As it is a peculiarity of these actions excited by these waters, that they all go on simultaneously—the activity of one, not entailing torpidity of the other, as is the case with the ordinary agents of our pharmacopœa, and notably, with the organs which seem vicariously to perform the functions of each other.

The third and last of the physiological actions of these waters consists in the increased demand and capacity for energetic alimentation, and whether this is due to specific tonic properties, or the antecedent actions already mentioned, need not now be considered. That these actions may be readily pressed to the development of pathological states may be readily inferred, from the common annoyance, and sometimes distress, from functional activity experienced by healthy, pleasure-seeking visitors.

I have now enumerated only the prompt, sensible effects of the Buffalo Lithia Waters, viz : exaltation of nerve force, and stimulated circulation, with diaphoresis, diuresis and purgation, and all these actions going on simultaneously. The professional reader will see at once that these are the means through which medical men, in all ages, have combated and overcome a wide circulation of diseases.

The ingestion and assimilation of external matters to its own structures, with the elimination of what has been rendered effete and hurtful, may be said to be the first conditions of animal life, and these vital functions are immediately reached by the actions above indicated.

The external cutaneous surface, with its internal prolongations to the cavities of all the hollow viscera with its vast glandular apparatus—these are the seats of these sensible actions. Let it be remembered that these same surfaces are the avenues through which morbific causes, whether from alterations of temperature or direct poisons, gain access to the animal economy ; also that the resulting structural and functional changes in these surfaces constitute the essence of the disease we seek to combat.

It would be beyond the limits designed for this paper to elaborate these ideas. But enough has been said to be suggestive of practical indications, and to explain on the basis of an accepted medical philosophy—the efficiency of these waters in *chronic engorgements* and *inflammations and atonies*—in the *removal of morbid accumulations and deposits*—and the *restoration to their normal physiological type, vitiated and suppressed secretions and excretions.*

I have now only treated of the palpable, sensible effects of these waters. But every physician is daily in the habit of instituting what is called *alterative medication*, by the gradual introduction of minute doses of medicinal agents into the system, and thus producing the most benign results. The testimonials in favor of the alterative actions of the Buffalo Lithia Waters are so full, that skepticism would argue greater mental weakness than credence. A large number of the astute medical observers from the frozen North to the Gulf—such as Wood, of Philadelphia, and Howard and Byrd, of Baltimore, and Beale and Hudson, of Virginia, and Jerman and Jones, of Carolina, and others of like attainments and capacity, furnish a body of experimental proofs on this point, which, even if not sustained by the positive results of the analytic chemist, would make it so much the worse for the chemistry.

But chemical analysis proves these waters to be rich in medicinal compounds. Their diuretic action secures efficient sewerage, thus relieving it of noxious products, and quieting irritations of the urinary passages, but also, their alkaline compounds counteract the acid diathesis so constantly present in rheumatism and gout, and by their chemical reactions secure, in soluble form, what would otherwise be retained in the shape of insoluble and hurtful deposits.

I call attention to the exceptionally large quantity of the carbonate of potash, and desire to record the declaration that during forty years of active professional life in a miasmatic region, I have derived more benefit in hepatic derangements, and malarial cachexies generally, from the gradual saturation of the system with this old fashioned "salts of tartar," than from any other treatment, regarding its alterative action as both safer and surer than that of the mercurial preparations.

The tonic and stimulating action of these waters is as well attested as any other of its effects. Drs. McGuire, Huston, Jones and others, describe this action as that of a "decided nerve tonic." This action is too prompt to be accounted for by improved nutrition, or the presence of a chalybeate. But the analysis reveals the fact that phosphorus is amongst its constituent elements, the great generator or nerve power, and especial pabulum of nerve tissue. True, it is put down in the quantitative analysis, as only "traces," but it should be remembered that this substance is so volatile as to be constantly changing form at ordinary temperatures, and as a medical agent it is used only in very minute doses, say from one-fortieth to one-twentieth of a grain—and, in these doses, it is "a powerful general stimulant with special tendency to the kidneys and genital organs," producing decidedly aphrodisiacal effects—and such are the well recognized effects of these waters. With the possession of these "nerve tonic properties," we readily account for their value in the great class of nervous diseases.

And so in chronic intermittent and remittent fevers, the disorders and cachexies which follow in their train, having been relieved in the manner already explained—the nervous system (the first to receive the impingement of the malarial poison) is fortified against its assaults by this "nerve tonic," whilst the work of a renovated nutrition is in steady progress.

It has never been intelligently claimed for these waters that they possessed any anti-periodic virtues in the sense in which Peruvian-bark is anti-periodic.

In the large class of what are called "female diseases," perhaps it is desirable to be somewhat explicit.

Of course these are limited to the ovaries, the uterus and their appendages; and, of course, no claim is set up for remedial powers in these waters over their *malignant* diseases and their structural changes.

But the functions of these organs are often interrupted by sympathy with other organs, or by faulty and deteriorated vitality in the general system, or alternations of temperature which declare their pathological state in the shape of *amenorrhœa*, *dysmenorrhœa*, *chlorosis*, or in passive and vitiated secretion. That these disorders should be often removed by an agent possessing the properties of these waters may be readily understood, especially when it is recollected that amongst these properties is that peculiar "nerve tonic" whose influence extends to the ovaries.

The *prophylactic* powers of the Buffalo Lithia Waters are very valuable in some conditions—as in procrastinating puberty, especially in those predisposed to constitutional diseases, in warding off the direful sequelæ of the *exanthematous* fevers, by keeping up healthy action on the new and callow secreting surfaces, and in some of the most distressing and dangerous diseases of pregnancy, by elimination of their exciting causes from the system.

There is one pathological condition in which I am sure these waters are contra-indicated. I mean in tubercular phthisis, after softening has taken place and hectic supervened. In many of these cases I have seen the circulation hurried, the cough more distressing, and colliquative sweats and diarrhœa increased to the speedy exhaustion of the patient.

In conclusion, the writer would say that he has long regarded these waters as a valuable agent of our *materia medica*, and *not* a panacea of indiscriminate application or universal efficacy. The described physiological actions are what takes place in a *healthy* person. Many invalids resort to these springs too much diseased to be benefited, until by intelligent care they are prepared to realize these effects.

---

**Strychnia as a Tonic.**—It would appear that strychnia or nux vomica is one of the most valuable tonics which we possess. When combined with nitro-hydrochloric acid it is perhaps one of the most efficient remedies that we can give for the debility which is so often noticed in warm weather, and when the ordinary tonics, such as gentian, columba, cascarilla, or quinine, do not produce the desired results, the addition of a little nux vomica or strychnia to them may give us the wished-for effect.—*Dr. Bronton, in Ec. Medical Journal.*

**Apomorphia as an Expectorant.**—Drawing a conclusion from the fact that ordinary emetics, when administered in small doses, generally act as expectorants, the director of the Heidelberg Polyclinic made experiments with small doses of apomorphia. It was found to greatly facilitate expectoration, and to quickly remove dry and sibilant rales. It was given in doses of one sixty-fourth to one-thirtieth grain in solution, the hydrochlorate being preferred.

